# Sex differences in brain transcriptomes of juvenile Cynomolgus macaques

**DOI:** 10.21203/rs.3.rs-3422091/v1

**Published:** 2023-11-20

**Authors:** Nadia Kabbej, Frederick J. Ashby, Alberto Riva, Paul D. Gamlin, Ronald J. Mande, Aishwarya Kunta, Courtney J. Rouse, Coy D. Heldermon

**Affiliations:** University of Florida; University of Florida; University of Florida; University of Alabama at Birmingham; University of Florida; University of Florida; University of Florida; University of Florida

**Keywords:** Sexual dimorphism, pre-pubertal, Cynomolgus Macaques, transcriptomics, translation, COVID-19, behavior, immunity

## Abstract

**Background:**

Behavioral, social, and physical characteristics are posited to distinguish the sexes, yet research on transcription-level sexual differences in the brain is limited. Here, we investigated sexually divergent brain transcriptomics in prepubertal cynomolgus macaques, a commonly used surrogate species to humans.

**Methods:**

A transcriptomic profile using RNA sequencing was generated for the temporal lobe, ventral midbrain, and cerebellum of 3 female and 3 male cynomolgus macaques previously treated with an Adeno-associated virus vector mix. Statistical analyses to determine differentially expressed protein-coding genes in all three lobes were conducted using DeSeq2 with a false discovery rate corrected P value of .05.

**Results:**

We identified target genes in the temporal lobe, ventral midbrain, and cerebellum with functions in translation, immunity, behavior, and neurological disorders that exhibited statistically significant sexually divergent expression.

**Conclusions:**

We provide potential mechanistic insights to the epidemiological differences observed between the sexes with regards to mental health and infectious diseases, such as COVID19. Our results provide pre-pubertal information on sexual differences in non-human primate brain transcriptomics and may provide insight to health disparities between the biological sexes in humans.

## Plain English Summary

In our study, we looked at the brains of young male and female monkeys to determine if their genes were being expressed differently. The expression of a gene is a measure of how often a gene’s function is being turned on or off in a cell. Like a light switch, a gene can be turned on to carry out its function or turned off if its function is not needed at that time. Using a technique called transcriptomics, we determined that young male and female monkeys expressed genes differently in three different brain regions: the temporal lobe, midbrain, and cerebellum. We were then able to relate multiple genes to larger biological processes using a technique called Gene Ontology, which groups genes with similar functions. Specifically, we found that males had more genes turned on with functions involved in the process of translation, social behavior such as dominance and aggression, and immune function regulation. It is known that certain diseases develop and present differently depending on the sex of an individual. For example, one gene in young male monkeys can delay the body’s immune response to COVID-19, which may be an explanation for why males have had worse outcomes in this disease. Other genes had functions involved in Alzheimer’s and mental health disorders. Our research can be used to better understand differences in gene expression between the sexes, because knowing these differences can help us develop studies that aim to prevent disease or develop treatments.

## Background

Non-human primates (NHP) are the closest phylogenetic surrogate model used in research to emulate the human brain^1^. Macaque models are critical to neurophysiology research because they share a significant overlap in structure and function with humans. Diverging from humans an estimated 26.8 million years ago, macaques share > 93% gene sequence similarity to that of humans^2,3^. Neocortical size is also comparable, consisting of 72% of the brain volume in Rhesus macaques and 80% in humans. Humans and macaques were also found to share structural homology in the rich club structures of their connectome, suggesting the route of information processing may be similar ^4^. These similarities in structure and higher-order brain function make macaques a robust and preferred model for human neuroscientific research.

Anatomical sexual dimorphism in the human brain has been described with relatively new neuroimaging techniques such as Magnetic Resonance Imaging (MRI). In humans, studies have found differences in cerebral volumes, white matter microstructure, neural connectivity, emotional processing, and visuospatial abilities ^5,6^. Literature specific to NHP is limited concerning sexual dimorphism and its characteristics. Studies using structural MRI in Rhesus macaques (MM) reported a larger overall brain volume and corpus callosum in males, and a larger striatum and hippocampus in females ^7,8^. A later study in Capuchin monkeys reported an enlarged hypothalamus in males and a larger cerebellum and visual cortex in females ^9^. These findings demonstrate a need for more in depth research methods to ascertain a complete understanding of sexual differences in macaques. Further, there is little research on NHP sexual differences in brain transcriptomics ^10^. Current research on the transcriptomics of macaques has revealed similarities to human expression profiles. Yin et al. compared the transcriptomic profile of a human brain with 52 brain regions in rhesus macaques and reported that humans and macaques have similar expression profiles in the thalamus, striatum, and cerebellar cortex,^11^ suggesting them as a valid model for transcriptomic comparisons. Tissue-specific microglial transcriptome in Rhesus macaques has also been explored, where genes exhibited comparable expression levels to humans ^12^. Macaque transcriptomic profiles, therefore, show reasonable translatability to humans and should provide important baseline data for researchers to use in future studies.

**Figure 1:**
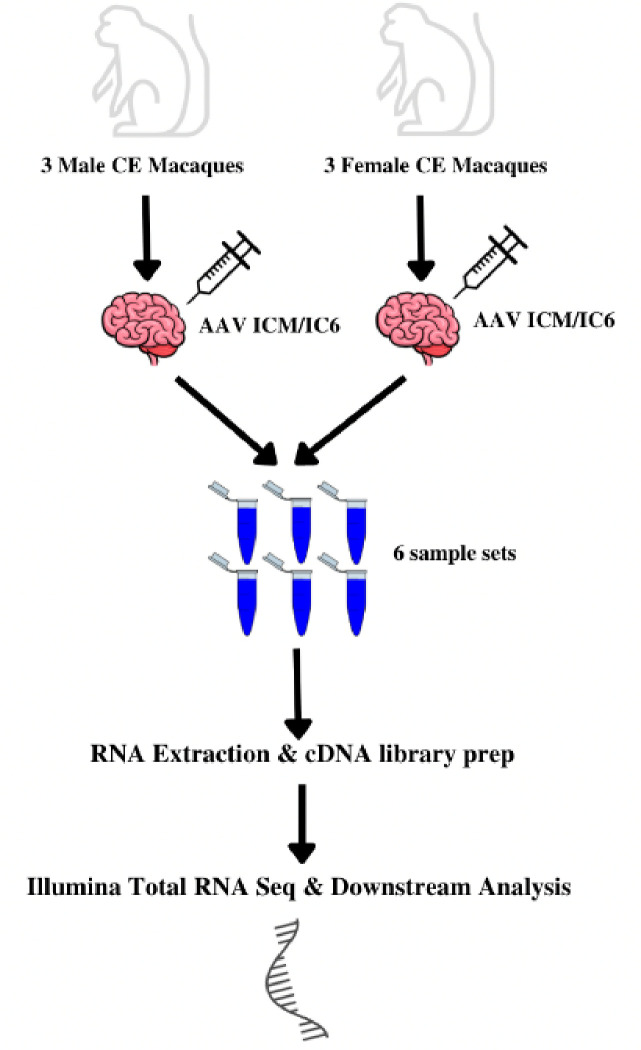
Experimental design

While considered distinct species, Cynomolgus (crab-eating; CE) macaque models (*M. fascicularis*) and Rhesus (MM) macaque (*M. mulatta*) have relatively small nucleotide divergence (0.34%). For comparison, nucleotide divergence within two subspecies of Rhesus macaques, Chinese (*M. mulatta lasiota*) and Indian (*M. mulatta mulatta*), were found to have 0.31% nucleotide divergence.^13^ Genomic comparison has also suggested Chinese Rhesus macaques may be more closely related to CE macaques than they are to Indian Rhesus macaques,^13^ adding complexity to the species distinction. In this study, we determine the degree of sex differences present in the transcriptomics of CE macaques. We carry out transcriptomic profiling on six young, prepubertal CE macaques previously treated with a barcoded Adeno-associated Virus (AAV) vector mix, using RNA-seq on samples obtained from the ventral midbrain, cerebellum, and temporal lobe (Fig. 1). We then conduct a male to female comparison of the expression levels of genes found in each brain region followed by a functional annotation analysis of processes involving these genes.

## Methods

### Animal Care, Brain Dissection, and Sample Processing

Six cynomolgus macaques, three males and three females, aged between 28 and 32 months, were procured from Bioculture Group (Glenmoor, PA), then housed and cared for at the University of Alabama in Birmingham. Animals were excluded that had pre-existing antibody titers to AAV vectors. In compliance with USDA’s Animal Welfare Act and Animal Welfare Regulations, the study methods for this project strictly adhered to the recommendations located in the Guide for the Care and Use of Laboratory Animals, as outlined by the National Institutes of Health. The Institutional Animal Care and Use Committee (IACUC) at the University of Alabama in Birmingham oversaw and approved all protocols and all animals were maintained in an AAALAC-accredited facility. Brain sample collection and animal euthanasia followed the standardized protocol previously described by Kondratov *et. al*. Briefly, anesthesia was induced with an intramuscular injection of ketamine (5–10 mg/kg) plus dexmedetomidine (0.015–0.02 mg/kg). Animals then received intravenous heparin sulfate (5,000–10,000 IU) to prevent blood clotting, followed by intravenous sodium pentobarbital to effect. Animals were perfused with 1.5 L of chilled (4°C) isotonic flush (0.9% sodium chloride/1.0% sodium nitrite in RNase-, DNase-free water). Immediately following euthanasia, the temporal lobe, ventral midbrain, and cerebellum were dissected by one trained neuroscientist knowledgeable on CE macaque anatomy, Dr. Ron Mandel, in the Department of Neuroscience at the University of Florida. For a more detailed description of dissection landmarks, see Kondratov *et. al*. Samples for the temporal lobe, ventral midbrain, and cerebellum were then stored in a phosphate buffer and placed in cryoprotectant prior to RNA extraction.

### RNA Extraction and cDNA Library Preparation

RNA isolation and purification were carried out by the Toxicology Core Facility at the University of Florida. For the temporal lobe, ventral midbrain, and cerebellum, RNA was isolated and prepared for extraction as previously described by Kondratov *et. al.* Total RNA for each sample was then extracted using a QIAGEN RNeasy^®^ Lipid Tissue kit (Cat #74804) according to the manufacturer’s instructions. Each sample’s RNA Integrity Number (RIN) was determined using the Agilent 2100 Bioanalyzer to ensure RIN for each sample was greater than seven.

Post-RNA extraction, a quality-controlled cDNA library was prepared and carried out by the University of Florida Interdisciplinary Center for Biotechnology Research (ICBR) in the NextGen DNA Sequencing (NGS) Core facility. Synthesis of cDNA libraries required 50 ng of RNA per sample, and cDNA synthesis reactions were performed using the NEBNext^®^ Strand-switch cDNA synthesis reagents (Cat #E6421). Reactions were first optimized using the RNA samples with the lowest RIN. Amplification reactions were performed using NEBNext^®^ High Fidelity 2X PCR Master Mix (Cat# M0541) at the following conditions: denaturation 98°C for 45 sec, then cycling 98°C for 10 sec, 62°C for 15 sec, 72°C for 3 min, final extension at 65°C for 5 min. Each cDNA product underwent quality control using the Invitrogen Qubit^®^ 3.0. Quality control was further ensured by testing every fourth sample using the Agilent 2200 Tape Station; at least 5 Tape Station runs were completed. Libraries were amplified for 14 rounds then sequenced on the Illumina NovaSeq6000. Overall, this generated 1,418,598,125-total reads with percent quality score greater than 30. These same optimized conditions were used to synthesize and amplify double-stranded cDNA for all samples.

### RNAseq Processing, Quality Control, and Normalization

Fastq files generated from the Illumina workflow underwent read-level quality control on original and trimmed reads using FAST QC (v0.11.4)^47^ and MultiQC.^48^ Short reads were trimmed using Trimmomatic (v0.36);^49^ about 95% of reads were retained after trimming. The reads were then aligned to the reference transcriptomes (ENSEMBL Macaca_fascicularis_6.0 and ENSEMBL Macaca_Mulatta_8.0) using STAR (v2.7.9a)^50^ and transcript abundance was quantified using RSEM (RSEM v1.3.1).^51^ Finally, differential expression analysis was performed using DESeq2^52^ with an FDR-corrected p-value of 0.05. The output files were then filtered to extract transcripts showing a 2.0-fold change in either direction.

### Gene Ontology Enrichment Analysis and Pathway Analysis

Previously identified significant gene sets from bulk RNAseq analysis in the temporal lobe, cerebellum, and ventral midbrain were analyzed using EnrichR. The 2021 Human Gene Ontology library was selected for the analysis; a NHP Gene Ontology library is not currently available. For the temporal lobe, significantly upregulated genes in males when compared to females were analyzed for terms overrepresented in Biological Processes (BP), Molecular Function (MF), Human Phenotype (HP) and Cellular Components (CC). For the cerebellum, significantly downregulated genes in males when compared to females were analyzed for BP, MF, and CC. The top 20 significant GO terms associated with each category (BP, MF, CC, HP) were then inputted into Revigo, a tool which groups similar GO terms together using Multidimensional Scaling to create graphical representations of term similarity. Pathway Enrichment Analysis for the temporal lobe was generated using EnrichR using the KEGG 2021 Human Pathway library.

## Results

The results reported are for protein-coding genes only. All gene expression analyses were performed as a male expression compared to female baseline. The temporal lobe was processed and analyzed separately, followed by the ventral midbrain and cerebellum, which were processed together. Due to considerable similarity between *M. mulatta* (MM) and *M. fascicularis* (CE), sequence reads were run against both transcriptome references to eliminate annotation bias. The CE macaque reference transcriptome left many statistically significant expression hits unannotated compared to the Rhesus macaque reference transcriptome, suggesting a potential annotation bias in the CE reference data. Thus, results between the CE and MM transcriptome references were non-redundantly combined to present the most thorough and complete analysis of target genes. For simplification, genes revealed by both references are discussed together with the CE taking priority; exact reads, genes, and figures for the MM and CE specific analysis are in supplemental material.

Total RNA-seq analysis on three male and three female macaques, aged between 28 and 32 months, revealed 84 statistically significant (p ≤ 0.05) sexually divergent gene expression levels in the temporal lobe. 78/84 genes were overexpressed in males and 6/84 were under expressed; 6 remained unidentifiable after a Basic Local Alignment Search Tool (BLAST) search was performed. Each sample had an average of 99 million raw reads (ranging from 92–105 million) with an average mapping rate of 75.43% (ranged 72.92 to 77.83%). A total of 14,218 protein-coding genes were detected. **Supplemental Table 1** lists all statistically significant differentially expressed genes in males compared to a female baseline within the temporal lobe of the brain. Of note, several interesting genes with functions related to translation were upregulated: Ribosomal Protein S4 Y-linked 1(RPS4Y1), Ribosomal Protein S4 Y-linked 2 (RPS4Y2), Eukaryotic Initiation Factor 1A Y-linked (EIF1AY), 60s Ribosomal Protein L37A (RPL37A), 28s Ribosomal Protein S35, mitochondrial (MRPS35), GTP Binding Protein 2 (GTPB2) and 60s Ribosomal Protein L23A RPL23A). The following genes related to behavior: Arginine Vasopressin (AVP), Dopamine D1 receptor (DRD1), and Cocaine Esterase (CES2), as well as genes related to immunity: MafB Zip transcription factor (MAFB) and Transcription factor AP-1 (JUN) had statistically significant (p < 0.05) differential expression between the sexes.

Pooled samples for the ventral midbrain and cerebellum had an average of 68 million raw reads (ranging 54–85 million for the ventral midbrain and 51–78 million for the cerebellum). The average mapping rate for the cerebellum was 81.82% (ranging 78.39–86.60%). In the Cerebellum, total RNA-seq revealed 33 sexually divergent protein coding genes; 19/33 were upregulated when compared to females and 14/33 were downregulated. **Supplemental Table 2** lists all statistically significant differentially expressed genes in males compared to a female baseline within the cerebellum region. Genes associated with mitochondrial metabolic processes such as Arginosuccinate Synthase 1 (ASS1) and Aldehyde Dehydrogenase 4 family member A1 (ALDH4A) were downregulated in the male macaque when compared to females.

The average mapping rate for the ventral midbrain was 74.82% (ranging 73.15–77.30%). In the ventral midbrain, total RNA-seq revealed 15 sexually divergent protein coding genes; 12/15 were upregulated and 3/15 were downregulated. **Supplemental Table 3** lists all statistically significant differentially expressed genes in males compared to a female baseline within the ventral midbrain region. Of note, Nephrocystin 3 (NPHP3), a gene integral to development, was overexpressed in the male macaques.

**Figure 2:**
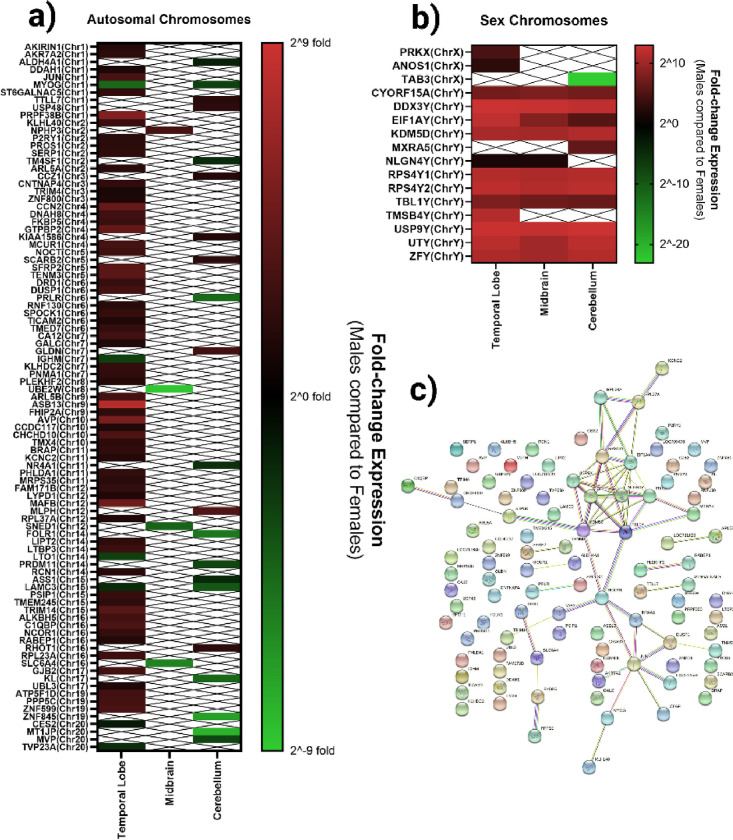
Differential Gene Expression in Males Compared to Females. Relative gene expression results in statistically significant differentially expressed genes in male macaques compared to female macaques is shown for autosomal chromosomes (a) and sex chromosomes b) on a log-base 2 scale. These genes were cumulatively analyzed by STRING for connected pathways (c).

Cumulative tissue expression for all three brain regions analyzed is shown in Fig. 2, with genes organized by chromosome of origin. Pathway analysis on differentially expressed genes between males and females was performed for individual brain regions and collectively using the STRING Database. Out of the 109 genes affected in total, 99 were annotated in the database when compared against the MM reference, and 51 were annotated when using the CE reference. Two pathways associated with gastrulation and zinc-finger gene regulation reached statistical significance in the cerebellum and ventral midbrain using the MM STRING reference (CL:20229 and CL:20223), while no pathways were identified against the CE STRING reference. These pathway clusters retained statistical significance when analyzing all brain regions simultaneously (p < 0.05). While both involve transcription factor regulation of gastrulation, CL:20223 also has strong involvement with oxidative stress, reactive oxygen species and starvation. To consider translatability to humans, the 109 genes were run against a *Homo sapiens* background. While 95 of these genes were annotated in the STRING database, no statistically significant pathways enriched.

**Figure 3:**
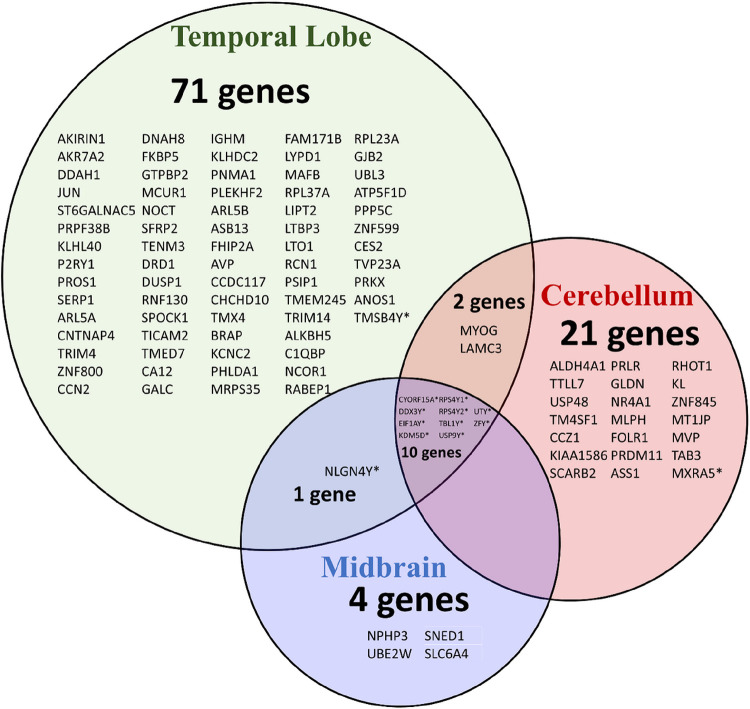
Venn Diagram of Differentially Expressed Genes in Male & Female Macaques. The Venn Diagram demonstrates the overlap between the statistically significant sexually divergent transcripts. Y-linked genes are annotated with an asterisk (*).

Figure 3 demonstrates a Venn diagram of the relationship between shared genes in the ventral midbrain, temporal lobe, and cerebellum to illustrate the overlap of sexually divergent, differentially expressed genes between the brain regions investigated. Not surprisingly, all protein-coding genes overlapping amongst the three lobes were Y-linked. In the ventral midbrain and temporal lobe, the gene Myogenin (MYOG), which is important to skeletal muscle development, and Laminin Subunit C3 (LAMC3), a constituent of the basement membrane, were both downregulated. Amongst the temporal lobe and cerebellum, the gene Neuroligin 4 Y-linked (NLGN4Y), which codes for a cell adhesion protein was downregulated. Its X chromosome counterpart has been associated with autism spectrum disorders^14^. The increased expression of NLGN4Y we report in young males, coupled with an absent increased expression of NLGN4X in females, coincides with previous transcriptomic data on sex differences in humans.^14–16^

**Figure 4:**
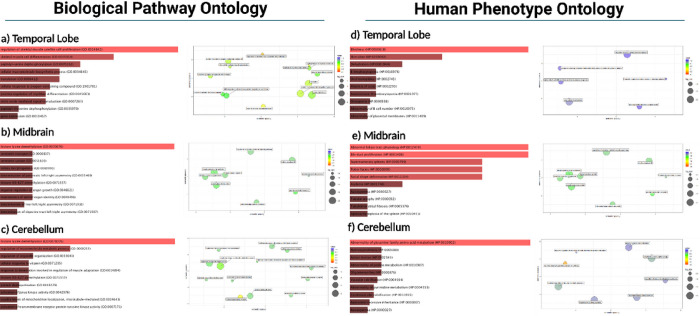
Biological Pathway and Human Phenotype Gene Ontology by Brain Location.

**Figure 5:**
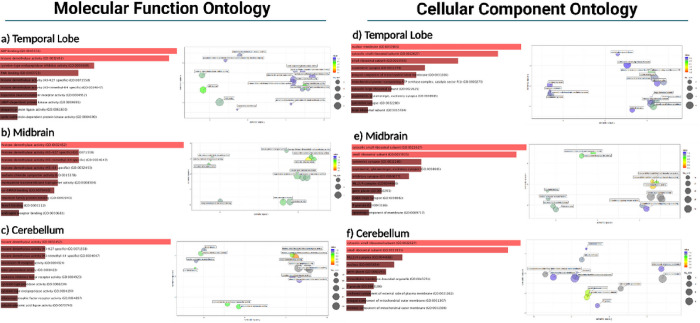
Molecular Function and Cellular Component Ontology for Sexually Divergent Transcripts.

For functional annotation of Gene Ontology (GO), enrichment analysis was performed using EnrichR for terms associated with Biological Processes (BP), Human Phenotype (HP) (Fig. 4), Molecular Function (MF) and Cellular Components (CC) (Fig. 5). Statistically significant (p < 0.05) upregulated and downregulated protein-coding genes in males when compared to females were analyzed to identify overrepresented terms. The below results are based on the GO libraries from the most up-to-date database (2021). GO terms for BP, HP, MF, and CC analyzed by EnrichR^17–19^ and found to be significantly upregulated from our gene list can be found in Fig. 4 and Fig. 5. Statistically significant terms generated from EnrichR were uploaded to Revigo^20,21^ to identify and visualize relationships between GO terms in each category in Fig. 4 and Fig. 5. Significant genes from the CE and Rhesus reference transcriptome were combined in the analysis and will be discussed together.

GO terms associated with the structural components and process of translation were significantly upregulated in the temporal lobe. GO terms such as translation (GO: 0006412), peptide biosynthetic process (GO: 0043043), SRP-dependent co-translational protein targeting to the membrane (GO:0006614), large ribosomal subunit (GO:0015934), and small ribosomal subunit (GO: 0015935) were statistically significant (p < 0.05).

Processes associated with transcriptional upregulation such as RNA binding (GO:0003723), histone lysine demethylation (GO: 0070076), histone H3-K27 demethylation (GO:0071557), and gene expression (GO:0010467) were upregulated as well as enzymatic processes associated with post-translational modifications such as phosphatase activity (GO: 0016791), peptidyl-serine dephosphorylation (GO: 0070262), peptidyl-threonine dephosphorylation (GO: 0035970), CAMP-dependent protein kinase activity (GO:0004691), and ubiquitin protein ligase activity (GO: 0061630).

Terms related to the regulation and function of the immune system such as regulation of complement activation (GO: 0030449), regulation of immune effector process (GO: 0002697), positive regulation of leukocyte chemotaxis (GO:0002690), and regulation of humoral immune response (GO: 0002920) were also overrepresented in the temporal lobe.

The following terms related to brain development and behavior were also upregulated in the temporal lobe of male macaques: regulation of androgen receptor signaling pathway (GO:0060765) and dopamine neurotransmitter receptor activity. The following synaptic cellular components were also upregulated: asymmetric synapse (GO: 0032279), asymmetric glutamatergic, excitatory synapse (GO: 0098985), symmetric synapse (GO: 0032280), and inhibitory synapse (GO:0060077).

Top statistically significant upregulated Gene Ontology terms for BP, HP, MF, and CC in the cerebellum demonstrated associations with: translation (GO: 0006412), peptide biosynthetic process (GO: 0043043), cytosolic small ribosomal subunit (GO: 0022627), small ribosomal subunit (GO: 0015935), histone demethylase activity (GO:0032452), and deubiquitinase activity (GO: 0101005).

The following are statistically significant downregulated terms relating to the Citric Acid Cycle and Urea Cycle in the ventral midbrain: aspartate metabolic process (GO:0006531), tricarboxylic acid metabolic process (GO: 0072350), arginine metabolic process (GO:0006525), citrulline metabolic process (GO:0000052), urea cycle (GO:0000050), and aspartate metabolic process (GO:0006531). The following terms relating to growth factors and receptor binding were also downregulated in the cerebellum of male macaques: cellular response to fibroblast growth factor stimulus (GO: 0044344), glucocorticoid receptor binding (GO:0035259), fibroblast growth factor binding (GO: 0017134), fibroblast growth factor receptor binding (GO: 0005104), growth factor receptor binding (GO: 0070851), and nuclear receptor binding (GO: 0016922).

Top significant upregulated Gene Ontology terms for BP, HP MF, and CC revealed terms associated with translation such as peptide biosynthetic process (GO: 0043043), translation (GO:0006412), RNA binding (GO: 0003723), cytosolic small ribosomal subunit (GO:0022627, and small ribosomal subunit (GO: 0015935), consistent with the temporal lobe and cerebellum.

The following similar terms associated with development and cell proliferation were also upregulated: kidney morphogenesis (GO:0060993), determination of pancreatic left/right asymmetry (GO: 0035469), determination of liver left/right asymmetry (GO: 0071910), determination of digestive tract left/right symmetry (GO: 0071907), ureter development (GO: 0072189), cardiac atrium development (GO:0003230), nuclear androgen receptor binding (GO: 0050681), and regulation of Wnt, planar cell polarity pathway (GO:2000095).

Statistically significant upregulated and downregulated genes were enriched through EnrichR Biological Pathway Ontology (a-c) and Human Phenotype Ontology (d-f), with the top 10 associated annotations shown ranked by −log_2_(p-value). These terms were uploaded to Revigo and graphically represented through principal component analysis based on strength of association and p-value.

Statistically significant upregulated and downregulated genes were enriched through EnrichR Molecular Function Ontology (a-c) and Cellular Component Ontology (d-f), with the top 10 associated annotations shown ranked by −log_2_(p-value). These terms were uploaded to Revigo and graphically represented through principal component analysis based on strength of association and p-value.

## Discussion

NHPs share many similarities to humans, yet research into the topic of sex differences within brain transcriptomics is not well understood. Genes that confer sex differences in brain development and disease progression could have important implications for human studies. Here, we add to the growing body of knowledge on sexual differences in prepubertal CE macaques, using total RNA-seq to evaluate transcriptomic sex differences in the temporal lobe, ventral midbrain, and cerebellum. Analyses of individual genes within each lobe, as well as Gene Set Enrichment Analyses, indicate many protein-coding genes involved in translation, immunity, social behavior, and development are expressed inherently differently between sexes.

We focused on transcriptomic sex differences, yet significant genes in all three lobes encoded for proteins that are necessary to proper translational function. Eukaryotic Initiation Factor 1A Y-linked (EIF1AY) was highly expressed in all three lobes in male macaques. EIF1AY encodes a protein that helps mediate the efficiency of translation initiation, aiding in both the transfer of a charged met-tRNA to the P site and the formation of the 40s pre-initiation complex ^22^. Previous research in humans comparing the expression of EIF1AY to its X chromosome homolog, EIF1AX, showed levels were relatively similar amongst tissues, apart from higher expression in the heart, skeletal muscle, spleen, pituitary gland, and blood ^23,24^. In the heart, a 5.8-fold increase in expression of EIF1AY was determined to be due to the loss of a target site for a microRNA called miR-1^23^. While miR-1 is widely expressed in the heart, about 60% of miR-1’s targets are distributed in brain tissue, therefore a relative deficiency of miR-1 activity to degrade EIF1AY transcripts in the brain could be one explanation for the increased relative expression we report in all three lobes for males compared to females^25^.

In addition, Ribosomal S4 Proteins, RPS4Y1 and RPS4Y2, which are necessary for the assembly of the 40s subunit during translation, were upregulated in all three lobes. RPS4Y1 has shown some level of expression in brain vestigial structures^26^. However, previous research on RPS4Y2’s expression in humans limits it to the prostate and testis; this is the first report, to our knowledge, that observes RPS4Y2 expression in the brain of an NHP^23,26^. Other genes involved in translation were specifically upregulated in the temporal lobe. For example, GTP Binding Protein 2 (GTPBP2), is a gene encoding for a protein that dissociates stalled ribosomes from mRNA to optimize translation elongation ^27–29^. Deficiencies in GTPB2 cause neurodegeneration in mice and a host of neurological issues in humans ^28,30^. The expression of genes such as EIF1AY, GTPBP2, RPS4Y1, and RPS4Y2 in the brain may point to male differences in translation initiation, elongation efficiency and regulation; more research is needed to confirm whether heightened expression corresponds to increased translation of these protein-coding genes.

Interesting genes related to immunity also exhibited sexually divergent expression in the temporal lobe. As a general rule, females tend to have stronger innate and adaptive immune responses than their male counterparts^31^. Thus, we were interested in exploring whether genes in our analysis related to immunity and disease could contribute to our understanding of sex-specific disease development and immunity in an NHP ^31^. MAF BZIP Transcription Factor B (MAFB), a gene highly upregulated in the temporal lobe of the male macaques, has been previously reported to play a sex-specific role in severe SARS-CoV-2 infectious disease (COVID) ^32,33^. Males are generally more susceptible to developing severe COVID, yet most discussions on susceptibility focus on co-morbidities, as opposed to sex-specific biology to explain this discrepancy ^34,35^. MAFB is a basic leucine zipper transcription factor that inhibits innate antiviral immune responses; it naturally binds to and blocks co-activator proteins from unnecessary induction of Type 1/*β* interferon (IFN) gene expression in uninfected cells ^36^. MAFB expression and subsequent IFN inhibition have been reported in plasmacytoid dendritic cells (pDC’s), microglia, and macrophages ^37,38^. MAFB’s connection to COVID emerges when a heightened baseline suppression of IFN may lead to a critical delay in the immune response during a viral infection. This delay is hypothesized to contribute to severe illness in males ^33^.

Evidence for MAFB’s role in severe COVID has been elucidated by its ability to inhibit Type 1 IFN activation downstream of the Toll-like receptor 7 (TLR7) pathway. TLR7 is one of the main pathways stimulated during a COVID infection. In addition, TLR7 escapes Chromosome-X inactivation, potentially leading to lower sensitivity of the TLR7 pathway in males prior to infection ^39^. Once infected with COVID, TLR7 and MAFB expression increase linearly over time, further contributing to an abnormal inflammatory response ^33^. A study on the transcriptomics of severe COVID patients found MAFB to be significantly upregulated in patients who were placed in the intensive care unit. Current research shows even mild COVID infections can cause neurological inflammation ^40^. Future research should focus on what role, if any, higher expression levels of MAFB in the brains of males play in this inflammation.

Genes linked to behavior and neurodevelopmental disorders were also identified in the male temporal lobe as sexually divergent; this may be important to our understanding of how neurological disorders develop and present differently amongst the sexes. Of note, the gene Arginine Vasopressin (AVP), a neuropeptide, was upregulated in male CE macaques. AVP has been previously reported to show greater expression in the hypothalamus of Rhesus macaques, but not in a sexually divergent fashion ^22^. Here, we are first to report that sexually divergent AVP expression occurs in the temporal lobe of prepubertal male CE macaques. Numerous studies related to AVP’s sex differences in mammals have focused specifically on the hypothalamus, where AVP has been shown to decrease social behavior and communication in males and increase aggression and attempts at establishing dominance ^41–44^. Anecdotally, AVP’s sexually divergent expression may provide insight into behavioral differences observed between males and females. The underlying signaling process for this increased expression and effect on behavior should be explored further in the future.

Another gene upregulated in the temporal lobe which has been linked to neurological disorders is the Dopamine Receptor D1 gene (DRD1). It codes for the most densely expressed dopamine receptor in the CNS, D1 ^45^. DRD1 has excitatory functions in memory, movement, impulse control, attention, and sleep ^45,46^. DRD1, and the dopaminergic system in general, has also been implicated in Alzheimer’s disease. Women account for 2/3 of Alzheimer’s disease diagnoses in the US, yet the sex-specific biological mechanisms into why this discrepancy occurs are limited. What is known is that decreases in dopamine receptors such as DRD1 create dysfunction in the dopamine system. A systematic review of the Dopaminergic system and Alzheimer’s found significantly lower levels of DRD1 in Alzheimer’s patients when compared to controls ^46^. Another study on Alzheimer’s disease patients reported lower expression of DRD1 specifically in the temporal lobe ^17^. This is the first study to report sexually divergent expression of DRD1 in the brain. Future studies should examine how DRD1 expression changes over time, and in other regions of the brain, for both sexes. Determining if higher DRD1 expression has a protective effect in the development of Alzheimer’s may provide insight into the higher incidence of the disease in females, as well as in developing treatment initiatives via Dopamine agonists.

This study was conducted alongside an AAV vector transduction comparison in support of animal use reduction. Animals were injected intracranially with a barcoded mix of AAV vectors; intracranial injection and/or AAV treatment may have influenced gene expression. However, we propose the gene expression differences we observed in the brain are primarily due to innate sex differences and not purely a response to the treatment of AAV since all animals received the same AAV injection mix and the proportion of RNA transcripts in these regions from the AAV luciferase/mApple reporter transgenes was less than 0.001%. To our knowledge, research into gene expression changes in the brain following the treatment of AAV vectors does not currently exist. It is possible that the expected immune response from the intracranial AAV injection could have exacerbated expression differences between males and females, especially in protein-coding genes that function to activate the immune system. However, it is worth noting that AAV at the doses injected does not typically generate strong immune reactions when compared to other gene therapy options such as adenoviral vectors.

The macaques in this study are pre-pubertal, therefore, the effects of age on gene expression cannot be ruled out. This study was also limited to three brain regions and had a focus on protein coding genes; transcriptomic profiles in more detailed structures of the brain would provide additional region-specific information on gene expression. Further, bulk RNAseq does not provide information of transcriptional differences between individual cells, nor does it detect small non-protein coding RNA such as miRNA and siRNA; these ncRNAs are important to transcriptional regulation such as splicing and silencing of mRNA. Most ncRNAs can be detected through single-cell RNAseq, but this analysis was not feasible due to cost constraints for this study. However, understanding transcriptomic changes within individual cells and cell types within individual brain regions would greatly improve this study. We propose a future spatio-temporal study that compares age, regions, and sex with total and single cell RNAseq; this would add to our understanding of gene expression, regulation, and brain development in an NHP.

### Perspectives and Significance:

Given the similarity in the transcriptomic profile of humans and macaques, the results of this study can be applied broadly to future research studies on CNS transcriptomics in humans and non-human primates. It may be a baseline to rule out genes which could pose sex-specific confounds to a study or inform researchers of sexual differences in their genes of interest. Most notably, mechanisms for sex differences in disease development, progression, and outcomes can be explored based on the target genes we identify here. The treatment of diseases with sex-specific etiology cannot be fully understood without an understanding of sexually divergent expression differences in health.

## Conclusions

Research using CE macaques continues to be a valuable model for human physiology and disease. Overall, this study adds to the growing body of literature on the presence of sexual differences in the brains of pre-pubertal non-human primates. Differences in genes associated with immunity, social behavior, aggression, and dominance were observed. Future research is necessary to confirm these findings and should focus on how sexually divergent expression profiles contribute to differences in the presentation of diseases in humans.

## Data Availability

All data generated or analyzed during this study are included in this published article [and its supplementary information files] and RNAseq data are available in the GEO database at accession number **GSE247236**.

## Highlights

- Behavior and physical characteristics separate the sexes, yet brain transcriptomic profiles may provide a more in-depth analysis of sexual dimorphism in individual gene expression.
- Using RNA sequencing, we conducted a male to female comparison of temporal lobe, cerebellum, and midbrain gene expression profiles in pre-pubertal Cynomolgus Macaques, a close surrogate species to humans.
- Sexually dimorphic gene expression (P<.05) was observed in all three lobes.
- Gene set enrichment analyses revealed the most widespread differences amongst the sexes were in biological processes associated with translation, immunity, and development.
- Individual genes showing sexually dimorphic expression have implications to human disease and development, especially in the context of sex-specific disease susceptibility to COVID-19.
